# Understanding the importance of weight management: a qualitative exploration of lived individual experiences

**DOI:** 10.1080/17482631.2024.2406099

**Published:** 2024-09-24

**Authors:** Arnel Esponilla Sarte, Edward Jay Mansarate Quinto

**Affiliations:** a Department of Psychology, School of Health Sciences, Mapua University Makati, Makati, Philippines; b Center for Teaching and Learning and Department of Psychology, Mapúa University Makati, Makati, Philippines

**Keywords:** Family support, health concerns, health consciousness, lived experiences, psychological motivation, weight management

## Abstract

**Purpose:**

To explore the lived experiences and motivations of individuals engaged in weight management, focusing on psychological, societal, health-related, and personal factors influencing their motivations.

**Methods:**

A descriptive exploratory approach, guided by the Self-Determination Theory (SDT), was followed and semi-structured interviews were conducted with ten adults actively involved in weight management. Thematic analysis identified key themes across four domains: (i) psychological motivation (extrinsic and intrinsic), (ii) societal influence (body image, social norms, media influence), (iii) health consciousness (priority of health, health concerns), and (iv) family support and past weight management experiences (learned habits, supportive environment).

**Results:**

The findings revealed a nuanced interplay of intrinsic and extrinsic motivations, societal expectations, health priorities, and the impact of family support on weight management. Aligning with SDT, the study emphasizes the role of autonomy, competence, and relatedness in shaping individual motivations for weight management.

**Conclusions:**

The study provides valuable insights for tailoring interventions aimed at enhancing well-being by addressing the psychological, societal, health-conscious, and familial factors that influence motivations in weight management.

## Introduction

Weight management is the process of maintaining a healthy weight through balanced diet and physical activity. It is an important aspect of health and well-being, as it can prevent or reduce the risk of various chronic diseases, such as obesity, diabetes, cardiovascular disease, and some cancers (Cai et al., 2022; Madigan et al., 2022). However, weight management is also influenced by a complex interplay of psychological, social, and environmental factors that affect an individual’s motivation, behaviour, and outcomes (CDC, 2023; National Institute of Diabetes and Digestive and Kidney Diseases, n.d.). Understanding these factors can help design more effective and personalized interventions for weight management, as well as improve the quality of life and self-esteem of individuals who struggle with their weight.

Despite the growing body of literature on weight management, there is a lack of qualitative research that explores the lived experiences and deep motives of individuals who engage in weight management. Most of the existing studies are quantitative and focus on the outcomes or correlates of weight management, such as weight change, body mass index, waist circumference, or biomarkers (Manore, 2015). However, these measures do not capture the subjective and contextual aspects of weight management, such as the emotions, beliefs, values, meanings, and challenges that individuals face in their weight management journey. Moreover, most of the studies adopt a deficit-based approach that emphasizes the barriers, problems, or failures of weight management, rather than the strengths, opportunities, or successes of individuals who manage their weight. Therefore, there is a need for a more holistic and positive perspective that acknowledges the diversity and complexity of weight management experiences and motives.

Autonomy is how people view their authority and freedom to choose and decide. Weight management requires understanding why people participate. Autonomy is crucial to this decision-making. It details how personal preferences, values, and choices affect motivation. Independent people are more likely to adopt and maintain weight management measures. Competence is one’s assessment of their ability to accomplish a task. Competence in weight management means having the confidence to make and maintain positive weight changes. Competence can illuminate the psychological determinants of motivation (Research Question 1) and health consciousness (Research Question 3). Understanding how people gain and maintain competence is crucial to devising weight management therapies that boost confidence. Relatedness is the need for human connection and meaningful interactions. Social norms, body image, family support, and past experiences affect weight management motivation, according to this study. It addresses Research Questions 2 and 4. This approach examines how relationships affect weight management. It considers interpersonal dynamics and support structures that might help or hinder success. Understanding how social factors affect motivation might help create interventions that leverage social support.

This involves addressing weight management emotions, attitudes, beliefs, meanings, and barriers. Understanding the causes is essential to designing effective weight management therapies that encourage long-term commitment. Thematic analysis, a qualitative method, supports SDT’s focus on subjective experiences and activity meaning. This helps uncover themes and patterns that indicate psychological desires for independence, skill, and connection. The study uses thematic analysis to show how important weight management is.

The aim of this study is to fill this gap by conducting a qualitative exploration of the lived individual experiences and deep motives of weight management. The main research question is: What deep motives do individuals have that make weight management important to them? This question is further divided into four sub-questions, each addressing a different dimension of weight management motives:

Central Research Question:

What deep motives do individuals have that make weight management important to them?

1. What psychological factors influence an individual’s motivation for weight management?
2. How does societal perception and body image impact an individual’s desire for weight management?
3. What role does health consciousness play in an individual’s decision to manage their weight?
4. How does an individual’s family support and past experiences in weight management shape their motivation for weight management?

To answer these questions, the researchers adopted a phenomenological approach that seeks to understand the essence and meaning of human experiences from the participants’ own perspectives. The researchers conducted semi-structured interviews with six adults who were actively involved in weight management, either for weight loss, weight gain or weight maintenance. The researchers used thematic analysis to identify the common themes and patterns that emerged from the data, as well as the variations and nuances that reflected the individual differences and contexts of the participants. The self-determination theory is used as a theoretical framework to interpret and explain the findings, as it provides a comprehensive and integrative model of human motivation and behaviour.

## Literature review

Weight management is a multifaceted area encompassing various dimensions, and a review of the related literature reveals a wealth of knowledge on the psychological, societal, health-conscious, and personal aspects influencing individuals’ motivations for weight management.

### Psychological factors

The interplay between psychological factors weight management is a rich area of research that underscores the importance of understanding the underlying mechanisms driving successful interventions. Self-Determination Theory (SDT), formulated by Deci and Ryan (2000), posits that motivation is deeply influenced by the satisfaction of three fundamental psychological needs: autonomy, competence, and relatedness. When these needs are met, individuals are more likely to experience intrinsic motivation, which is essential for long-term adherence to weight management behaviours. Teixeira et al. (2011) reinforce this notion, highlighting that autonomous motivation, where individuals engage in behaviours out of personal interest and values rather than external pressure, is crucial for sustainable weight loss and maintenance. Silva et al. (2011) demonstrated that interventions promoting autonomy-supportive environments, where individuals felt their choices were respected and they were encouraged to take responsibility for their health, led to more significant weight loss and better psychological outcomes compared to control groups. This emphasizes the importance of creating supportive contexts that foster intrinsic motivation for weight management.

Self-efficacy, introduced by Bandura (1977), refers to an individual’s belief in their capability to execute behaviours necessary to produce specific performance attainments. This concept is critical in weight management, as higher self-efficacy is associated with greater confidence in one’s ability to adhere to diet and exercise regimens. For instance, Linde et al. (2006) found that individuals with higher self-efficacy were more successful in initiating and maintaining weight loss. Moreover, interventions aimed at increasing self-efficacy, such as goal setting, self-monitoring, and providing mastery experiences, have been shown to be effective. Napolitano et al. (2013), which demonstrated that participants who received self-efficacy-enhancing strategies during a weight loss programme achieved better outcomes compared to those who did not.

Locus of control, as conceptualized by Rotter (1966), refers to an individual’s perception of control over the events in their life. Those with an internal locus of control believe that outcomes result from their own actions, while those with an external locus of control attribute outcomes to external factors. In the context of weight management, an internal locus of control is associated with greater motivation and proactive health behaviours. Wallston et al. (1978) found that individuals with a higher internal locus of control were more likely to engage in health-promoting behaviours and achieve better weight management outcomes. This suggests that fostering an internal locus of control through cognitive-behavioural interventions can enhance motivation and success in weight management.

Social support is another crucial factor influencing motivation in weight management. Research indicates that support from family, friends, and peers can significantly impact an individual’s ability to adhere to weight management programmes. Wing and Jeffery (1999) found that participants who engaged in weight loss programmes with friends or family members had better outcomes than those who participated alone, highlighting the motivational benefits of a supportive social network. Emotional regulation, the ability to manage and respond to emotional experiences, also plays a vital role in weight management. Poor emotional regulation can lead to emotional eating, where individuals use food to cope with negative emotions, undermining weight loss efforts. Canetti et al. (2002) found that interventions targeting emotional regulation skills, such as mindfulness and stress management, were effective in reducing emotional eating and promoting weight loss.

The literature underscores the multifaceted nature of motivation in weight management, with psychological factors such as autonomy, self-efficacy, locus of control, social support, and emotional regulation playing pivotal roles. The Self-Determination Theory (SDT) provides a comprehensive framework for understanding these dynamics, while concepts like self-efficacy and locus of control offer specific insights into how motivation can be enhanced. Effective weight management interventions should thus consider these psychological dimensions to foster intrinsic motivation and support long-term success.

### Societal factors

The societal standards and perceptions regarding body image play a pivotal role in influencing individuals’ motivations for weight management. The significant impact of media portrayal, societal expectations, and cultural ideals contributes to the formation of body dissatisfaction, as highlighted by Perloff (2014). This dissatisfaction with one’s body is closely linked to the motivation to partake in weight management behaviours, encompassing activities like dieting and exercise, as outlined by Neumark-Sztainer et al. (2006). The Social Comparison Theory, originally proposed by Festinger (1954), is frequently invoked to elucidate how individuals assess their own bodies in comparison to others. This process of social comparison significantly influences their motivation to conform to societal norms, as discussed by Myers and Crowther (2009). In essence, the way people perceive their bodies in relation to societal ideals serves as a powerful motivator, steering their efforts towards conforming to prevailing norms of attractiveness and acceptability.

Recent research further substantiates the role of societal standards in shaping body image and weight management behaviours. Tiggermann and Slater (2013) found that exposure to idealized body images in the media is strongly correlated with body dissatisfaction and the desire to lose weight, particularly among young women. This suggests that the media’s portrayal of thinness as the ideal body type exerts substantial pressure on individuals to engage in weight management practices. Additionally, Rodgers et al. (2013) emphasized the role of social media in perpetuating unrealistic body standards. Their study indicated that frequent use of image-centric social media platforms, such as Instagram, is associated with increased body dissatisfaction and unhealthy weight control behaviours. This is corroborated by the findings of Fardouly et al. (2015), who reported that social media comparisons often lead to negative body image and a heightened drive for thinness among adolescents and young adults.

The relationship between societal perceptions and individual motivations is further complicated by cultural factors. According to Swami (2015), cultural differences significantly influence body image perceptions and weight management behaviours. For example, in some non-Western cultures, larger body sizes are often associated with health and prosperity, contrasting sharply with Western ideals of thinness. This highlights the importance of considering cultural context when examining the impact of societal standards on body image and weight management. Furthermore, the internalization of societal ideals can lead to harmful psychological effects. Grabe et al. (2008) conducted a meta-analysis revealing that internalizing media messages about the ideal body is linked to increased levels of body dissatisfaction, eating disorders, and depression. This underscores the need for interventions that promote body positivity and challenge unrealistic body standards.

To address these issues, promoting a more inclusive and realistic understanding of diverse body shapes and sizes is crucial. Emphasizing individual health and well-being over rigid societal standards may contribute to a more positive and sustainable approach to weight management motivations. Neumark-Sztainer et al. (2006) advocate for a health-centred approach that focuses on healthy behaviours rather than weight loss, suggesting that this could mitigate the adverse effects of body dissatisfaction and promote long-term well-being.

In conclusion, the intricate relationship between societal perceptions and individual motivations for weight management underscores the significant influence of societal standards on body image. Addressing these societal influences by fostering body positivity and emphasizing health over appearance can help individuals develop a healthier relationship with their bodies and weight management behaviours.

### Health consciousness

Health consciousness, characterized by an individual’s awareness and concern for their well-being (Hong, 2009), is a significant factor influencing decisions related to weight management. The concept of health consciousness plays a crucial role in motivating individuals to take proactive steps in maintaining a healthy weight. According to the Health Belief Model proposed by Rosenstock (1974), individuals are motivated to adopt weight management behaviours as a preventive measure when they perceive themselves as susceptible to health risks.

The statements suggest that the perceived susceptibility to health risks serves as a key motivator for individuals to engage in actions that contribute to weight management. This awareness of potential health consequences can act as a powerful driving force, prompting individuals to make choices that align with maintaining a healthy weight. This preventive mindset becomes particularly relevant when considering the potential long-term health implications associated with weight-related issues.

Moreover, research conducted by Puhl and Heuer (2009) supports the idea that health-conscious individuals are more likely to sustain their efforts in weight management. This indicates that a heightened awareness of one’s health may contribute to a more committed and enduring commitment to weight-related behaviours, such as maintaining a balanced diet, engaging in regular physical activity, and making informed lifestyle choices. A more recent study by Y. Wang et al. (2022) found that health consciousness significantly predicted dietary self-regulation, which in turn was associated with better weight control outcomes. This underscores the importance of fostering health consciousness as a means of enhancing dietary behaviours that support weight management. Recent research supports this view, indicating that individuals with a high degree of health consciousness are more likely to engage in preventive health behaviours, including those related to weight management (Jayanti & Burns, 1998). This heightened awareness not only encourages healthier lifestyle choices but also enhances the individual’s ability to sustain these behaviours over time.

Rogerson et al. (2016) explored how weight loss was experienced as an enduring challenge, where factors that assisted weight loss were developed and experienced dichotomously to factors that hindered it. This highlights the complexity of weight management and the need for health professionals to better understand the day-to-day challenges of individuals aiming to lose weight. Madigan et al. (2022) conducted a systematic review and meta-analysis of randomized controlled trials to examine the effectiveness of behavioural weight management interventions for adults with obesity delivered in primary care. They found that these interventions resulted in effective weight loss and reduction in waist circumference. This suggests that health-conscious individuals who engage in behavioural weight management interventions can achieve significant improvements in their weight status.

In another research by Ingels et al. (2018), weight management may improve more than just the physical health. In addition to weight loss, participants had hopes of enhanced health, fitness, and mood, as well as receiving support and cultivating new behaviours. Participants deliberated on evaluating their achievements by prioritizing weight reduction and alterations to their physique, enhanced well-being, emotional state, and physical fitness, establishment of new routines, and acquiring supplementary assistance.

In a broader context, fostering health consciousness can be seen as a valuable approach in promoting holistic well-being. Encouraging individuals to be mindful of their health not only addresses weight management concerns but also promotes a proactive and preventive mindset that extends to various aspects of a person’s overall health. Thus, integrating health consciousness into decision-making processes can be a key component of fostering a sustained commitment to weight management and over well-being.

### Family support and past experiences from weight management

An individual’s historical background and past encounters play a crucial role in shaping their motivation for weight management. The outcomes, whether successes or setbacks, from previous attempts at managing weight significantly influence an individual’s current approach to their weight management journey. Wadden et al. (1983) emphasized that understanding these past experiences can provide valuable insights into tailoring effective strategies for the present. Further, Ogden (2000) found that individuals who had experienced repeated failures in weight loss often developed a sense of learned helplessness, making them less likely to engage in new weight management efforts. Conversely, individuals with a history of successful weight loss attempts often approached new weight management programmes with greater confidence and determination (Wing & Phelan, 2005).

Research findings underscore the impactful role of family support in influencing weight management outcomes. M. L. Wang et al. (2014) demonstrated that individuals with higher levels of family support for physical activity exhibited an average weight decrease of 0.032 kg for each unit increase in support scores over a 24-month period. This highlights the tangible influence of family dynamics on weight-related efforts. Additionally, the presence of companionship from family members and friends has been linked to elevated levels of physical activity among adults (Trost et al., 2002). Qualitative research further indicates that these companions play essential roles by offering motivational, social, facilitative, and instructional support for physical activity (Ball et al., 2010).

Moreover, a meta-analysis by Black et al. (2010) found that family-based interventions were more effective in promoting weight loss among overweight and obese children and adolescents compared to individual-based interventions. This underscores the importance of a supportive family environment in fostering healthy behaviours and achieving weight management goals. Similarly, Gorin et al. (2008) highlighted that adults who received family support in their weight loss efforts reported better adherence to dietary and exercise recommendations, resulting in greater weight loss compared to those who lacked such support.

These findings collectively suggest that, particularly among adults, targeting family support for physical activity can be a crucial focus for interventions aimed at promoting healthy weight management. Recognizing and fostering a supportive environment within the family unit can significantly impact an individual’s motivation and success in weight management endeavours. This approach acknowledges the multifaceted nature of support, encompassing not only motivational aspects but also social, facilitative, and instructional dimensions (Sallis et al., 2009). Additionally, Epstein et al. (2007) found that family-based behavioural treatment resulted in significant weight loss and improved physical activity levels among children and their parents, highlighting the intergenerational benefits of such interventions.

In summary, acknowledging and learning from past weight management experiences, coupled with recognizing the influential role of family support, provides a comprehensive perspective for designing effective interventions. By addressing these elements, interventions can be more personalized and tailored to the individual’s unique history and social context, fostering a holistic approach to healthy weight management.

### Research design

The selected research design for this study is a descriptive exploratory approach, which is a qualitative research method that seeks to offer a thorough depiction and comprehension of a specific phenomenon by investigating its diverse aspects and situations (Polit & Beck, 2017). This design is particularly suitable for investigating obscure or intricate matters, providing a comprehensive and intricate depiction of the subject matter (Creswell & Poth, 2016). In this instance, the implementation of a descriptive exploratory design will enable a comprehensive examination of the motives and experiences associated with weight control. This approach will effectively capture the complex and subtle elements of these experiences as perceived by the participants.

Descriptive exploratory research is highly useful for investigations aiming to collect comprehensive and extensive data about a phenomenon to discern patterns, themes, and insights (Sandelowski, 2000). This methodology enables researchers to maintain receptiveness towards uncovering novel understandings and revelations that arise from the data, rendering it a suitable option for investigating the intricate and individualistic aspects of weight control motivations and experiences. This study uses a descriptive exploratory methodology to identify and explain the different elements that impact individuals’ weight management journeys. The goal is to establish a basic understanding that can be used to guide future research and practical interventions in this field.

### Participants and sampling technique

The participants in this study consist of 10 young adults from ages 19 to 23 years old actively engaged in weight management, either aiming for weight loss, weight gain, or weight maintenance. The inclusion criteria for participants involve individuals who have a history of involvement in weight management practices and are willing to share their lived experiences and deep motives related to this process.

To ensure the selection of appropriate participants for this study, purposeful sampling will be employed. The target participants are individuals from various universities, including student-athletes, regular students, and working students. Aside from the settings that the participants are from, they are also reached by networking through recommendation of common acquaintances or classmates in the master’s class. The primary criterion for inclusion in the study is active engagement in weight management practices. This includes activities such as monitoring diet, participating in physical exercise, or employing other strategies aimed at maintaining or achieving a specific weight. Potential participants will be excluded if, upon initial inquiry, they are not currently and actively managing their weight. This criterion ensures that the study focuses on individuals who are presently engaged in weight management, which is essential for the relevance and validity of the research findings (Palinkas et al., 2013; Patton, 2014). Participants will then be selected purposefully to ensure that individuals with diverse backgrounds, experiences, and motivations related to weight management are included. This method allows the researchers to capture a rich and varied range of perspectives. Data saturated on the tenth interview.

Efforts are made to include participants from various demographic backgrounds, including age, and gender. This ensures a more comprehensive understanding of the factors influencing weight management motivations (see Table 1).

**Table 1.** Demographic profile of participants.

Participants	Gender	Age	Classification	Weight Management Goal
Swimming Throwing Events Basketball Body Building Track and Field Body Goals Fit Tracker Pilates Shuawawa Angela	Male Male Male Male Male Female Female Female Female Female	22 23 23 19 23 20 22 23 21 22	Student Athlete Student Athlete Student Athlete Working Student Student Athlete Student Student Newly Graduate Student Student	Maintain Maintain Lose Maintain Maintain Lose Lose Lose Lose Gain

The table above presents that the participants are given code names that they want to use in this research, as informed consent was given before the study began. There are five male participants and five female participants. Four of the male participants has maintain as their weight management goal, while one has lose as of the weight management goal. For female participants, four of them has lose for their weight management goal while one is gain as the weight management. Participants were given extensive details regarding the essence, objective, and possible consequences of the research. Obtaining informed consent from every participant, assuring their comprehensive comprehension of their voluntary participation, the utilization of their personal data, and the entitlement to withdraw from the study at any time without facing any repercussions.

### Data collection technique

The chosen data collection technique for this research is semi-structured interviews. Semi-structured interviews offer a flexible and dynamic approach, allowing the researchers to explore the lived experiences and deep motives of individuals engaged in weight management in a comprehensive manner. This technique enables a balance between the guidance provided by a set of predetermined questions and the flexibility to delve deeper into participants’ responses, capturing rich qualitative data. The use of semi-structured interviews aligns with the phenomenological approach adopted in this study, aiming to understand the essence and meaning of individuals’ experiences (Creswell & Poth, 2016).

Semi-structured interviews provide a balance between structure and flexibility, allowing for a nuanced exploration of participants’ motivations for weight management in alignment with the study’s focus on psychological, societal, health-conscious, and personal factors. This balance ensures that while the interviews remain focused on key areas of interest, they also allow for the discovery of unanticipated insights that are crucial for a thorough phenomenological understanding (Smith & Osborn, 2015). While it is true that focus group discussions (FGDs) are also a common technique for data collection in such studies, interviews are considered equally trustworthy data sources (Kvale & Brinkmann, 2009). The individual nature of semi-structured interviews is particularly advantageous for this research as it allows participants to share personal and potentially sensitive information about their weight management experiences in a confidential setting, which might not be as easily disclosed in a group setting (Gill et al., 2008). This individualized approach aligns with the core aim of phenomenology to deeply understand personal lived experiences and the meanings individuals ascribe to them (Van Manen, 2016).

Thus, semi-structured interviews are well-suited for this study, providing the necessary depth and flexibility to uncover the intricate motivations behind weight management, capturing a comprehensive picture that aligns with the phenomenological approach. After preparing the semi-structured interview questions and methodology, the researchers obtained ethics clearance and permission from the Institutional Ethics Committee of their university (FM-RC-27-06-28).

Questions that were only partially structured were asked of the participants during the interviews to rapidly uncover themes and patterns.

(1) **What psychological factors influence an individual’s motivation for weight management?**
  1. Can you describe any personal beliefs or attitudes that motivate you to manage your weight?
  2. Can you describe how your mindset affect your weight management efforts?
  3. How does your personal goals and aspirations relate to your weight management?
(2) **How does societal perception and body image impact an individual’s desire for weight management?**
  1. Can you share how societal standards or expectations about body image influence your weight management goals?
  2. What are some examples of how social norms or expectations have influenced your motivation to manage your weight?
  3. How do you feel about the way the media depicts body image and how does it impact your weight management strategies?
(3) **What role does health consciousness play in an individual’s decision to manage their weight?**
  1. How important is your health in your decision to manage your weight?
  2. Can you share how your awareness of health issues related to weight has influenced your weight management?
  3. Can share any experiences of specific health concerns that have prompted you to manage your weight?
(4) **How does an individual’s personal history and experiences shape their motivation for weight management?**
  1. Can you share any past experiences that have shaped your current approach to weight management?
  2. How have your family’s attitudes and behaviours towards weight management influenced your own?

*(Please be advised that the researchers employed queries such as “Could you provide an example?” and “Could you elaborate on that?” to elicit more comprehensive information when necessary.)*

### Data analysis

In order to recognize recurring topics and patterns within the interview data, the method of thematic analysis, as described by Braun and Clarke (2006), was successfully implemented. The use of this qualitative methodology made it possible to conduct an in-depth investigation of the lived experiences and motives of the participants about weight control.

#### First coding

At first, the transcripts were coded using MAXQDA to find important words, phrases, and feelings linked to weight management. The goal of this method is to get a full picture of the dataset.

#### Finding the theme

After the original coding, the codes were put together to look for themes and patterns. Themes were found by looking for patterns that showed up repeatedly and making links that made sense. Themes that are not too relevant and have very few codes to represent them were still showed but as candidate themes.

#### Amount of saturation and adherence

The analysis went on until there was no more data to look at. This made sure that no new themes came up and that existing themes were consistently shown in all the participant interview answers.

## Results

The present research explores what are the deep motives that individuals have that make weight management important to them. Revealing 4 distinct themes that surfaced after the coding and analysis of data from each research interviews based on the research questions is done.

### Main themes

1. **Psychological Motivation (Theme)**
  1. *Extrinsic Motivation (Code)*
  2. *Intrinsic Motivation (Code)*
2. **Societal Influence (Theme)**
  *Body Image (Code)*
  *Social Norms (Code)*
  *Media Influence (Code)*
3. **Health Consciousness (Theme)**
  *Priority of health (Code)*
  *Health Concerns (Code)*
4. **Family Support and Past weight management experiences (Theme)**
  *Learned Habits (Code)*
  *Supportive Environment (Code)*

### Candidate themes

There are three candidate themes that emerged during the data analysis. These themes demonstrated a potential pattern or topic identified during data analysis these themes exhibited initial relevance or significance but lacks sufficient evidence to be classified as a primary theme. These themes emerge from the coding process and may include a few instances or codes, indicating their presence in the data. However, they do not demonstrate the depth, frequency, or robustness required to be considered main themes. Regardless, these themes have been present in the interviews of some of the participants and are naturally mentioned. These themes may also indicate other factors influencing weight management; however, caution must be taken in considering these candidate themes as, in this paper’s data they were not as recurring as the actual themes.

- **Negative Self-Comparison**

I think especially sometimes… My sister is a model, right? So, it influenced… Before, it feels weird that I’m the small one and then I’m the chubby one. It feels weird. And then, it makes me pressured to keep up. (Pilates, 23)

In the case of Pilates, the interview excerpt illustrates the theme of negative self-comparison. Pilates compares herself unfavourably to her sister, a model, which leads to feelings of pressure and discomfort about her own body size and shape. This negative self-comparison acts as a motivation for Pilates to manage her weight, as she strives to reduce the perceived gap between her own body and her sister’s. However, while this can lead to healthier behaviours, it is important to note that it can also result in unhealthy practices if the pressure becomes too intense. Furthermore, it can have a significant impact on mental health, potentially leading to low self-esteem, body dissatisfaction, and even eating disorders. Therefore, while negative self-comparison can act as a motivator, itis crucial for Pilates to approach weight management in a balanced and healthy way.

- **Unsupportive Environment**

But when I started gaining weight and muscle and bulking up, that’s when they started making little comments that discouraged me from working out, as if it was too much. (Angela, 22)

They’re hurtful. Until now, I’d say, I don’t have that much self-esteem. I guess right now, I’m learning just to disregard their comments if itis a lack. Especially the comparison. Like, if you’re thinner before, I’m thinner during this time and stuff at your age. (Pilates, 23)

The interview excerpts from Angela and Pilates both highlight the theme of an unsupportive environment. Angela mentions that as she started gaining weight and muscle, people around her began making discouraging comments about her workout routine. Similarly, Pilates talks about hurtful comments and comparisons that have negatively impacted her self-esteem. An unsupportive environment can be a significant factor in weight management motivation. When individuals face negative comments or lack of support from their environment, it can create a sense of pressure or obligation to conform to certain standards or expectations. In the case of Angela and Pilates, the unsupportive comments and comparisons have motivated them to manage their weight, albeit in a stressful and potentially unhealthy way

- **Self-Acceptance**

For me, societal standards or expectations doesn’t have an effect for me because as long as you love yourself and as long as you see yourself in a specific weight management goal, you could gradually go through that and yeah, so the standards don’t affect me for my weight management goals. (Basketball, 23)

Yeah. Right now, I’d say, even if I’m not in the best way right now, I’m trying to learn how to love and accept myself because it is so sad that I can’t choose to be happy now. Like, I have to choose to be happy later which shouldn’t be the case. (Pilates, 23)

Ever since I learned how to tune them out, I learned that itis better if the approval comes from within, that I have to learn to love myself and undo years’ worth of comments that damaged how I perceive my own body and thus myself, because I don’t want to feel like a stranger in my own body anymore. (Angela, 22)

The interview excerpts from Basketball, Pilates, and Angela all highlight the theme of self-acceptance. Basketball expresses that societal standards don’t affect their weight management goals because they prioritize self-love and personal goals. Pilates, despite not being in her best state, is learning to love and accept herself, choosing happiness now rather than later. Angela has learned to tune out negative comments and seeks approval from within, aiming to love herself and undo the damage caused by years of negative comments. Self-acceptance can be a significant motivator in weight management. When individuals accept and love themselves as they are, they are more likely to set healthy and realistic weight management goals that are based on personal well-being rather than societal standards or external comments. In the case of Basketball, Pilates, and Angela, self-acceptance has become a driving force in their weight management journey, motivating them to maintain a healthy weight that feels right for them.

### Main themes

#### Psychological motivation

In relation with the first research question “What psychological factors influence an individual’s motivation for weight management?” the theme psychological motivations emerged which then established two codes that indicates that psychological factors that come from both extrinsic and intrinsic motivations are deep motives for weight management.

Extrinsic motivation:

I just want to fit in because I’m also a part of the team that needs to lose weight and be in good condition to perform well. (Track and Field, 23)

Yes. Since our event, our weight doesn’t really matter. The more we gain weight, the more we get stronger. (Throwing Events, 23)

If I achieve the body that I want and maintain my weight in that way, people will accept me more, something like that. (Fit Tracker, 22)

Yeah, I guess it is a big thing. Sometimes, it makes me wish that I can be a bit more lean, a bit more muscular (Pilates, 23)

Track and Field is motivated to lose weight and be in good condition to fit in with their team, seeking the acceptance and approval of the team as an external reward. Throwing Events believes that gaining weight will make them stronger and presumably more successful in their event, with improved performance and possible recognition for their strength serving as external rewards. Fit Tracker user is motivated to achieve a certain body type to be more accepted by people, with the acceptance and positive recognition from others serving as the external reward. Lastly, Pilates desires to be leaner and more muscular, driven by the potential admiration from others, which is an external reward. These cases all demonstrate extrinsic motivation in relation to weight management.

Intrinsic motivation:

I disciplined myself when it comes to weight management because I lack motivation, as motivation is just a fleeting feeling, but discipline is enduring. (Body Building, 19)

For me, I believe that everything is in the mind. If you want to achieve something, you really need to sacrifice when it comes to it. So, my mindset is always, if not now, then when? So, it is very timely when it comes to maintaining my physique. (Swimming, 22)

I feel the best when I’m confident with my weight and not just that—it also gives me strength and power. That’s why I really like managing my weight and my workout activities. (Shuawawa, 21)

I feel like if I don’t do this (working out, gaining weight for muscles), I’ll forever be stuck in the box of expectations of how other people (namely my mom, haha, sorry, I really have mommy issues) want me to look—and I can’t let myself be constantly weighed down by that. (Angela, 22)

The individuals are motivated by their own internal desires and commitments, rather than external rewards or recognition. This is the essence of intrinsic motivation. Body Building talks about disciplining themselves in the context of weight management. This shows that Body Building is internally driven to maintain their weight, not because of external factors, but because they have made a personal commitment to themselves. Swimming believes that everything is in the mind and that achieving something requires sacrifice. Their mindset of “if not now, then when?” shows a strong internal drive to maintain their physique. They are not waiting for external factors to prompt them into action, but are instead motivated by their own desire to achieve their goals. This proactive approach is another example of intrinsic motivation. Shuawawa finds personal satisfaction, strength, and power in managing their weight and workout activities, which are all internal rewards. Lastly, Angela is motivated to work out and gain muscle weight to break free from others’ expectations and avoid feeling weighed down, which is a form of personal liberation, an internal reward. These individuals are all driven by intrinsic motivation as they pursue weight management for personal reasons and rewards.

## Societal influence

In relation to the second research question “How does societal perception and body image impact an individual’s desire for weight management?”The theme societal influence emerged which then established three codes (Body Image, Social Norms, and Media Influence) which indicates that these three are deep motives for weight management.

Body image:

So, for example, when I was training and I got sick, I had teammates whose minds were like old fashioned. They told me, you’ve lost weight. But I never once criticized their physique. (Swimming, 22)

With how the media depicts body image I think that its negative for many people as well as for myself. I get insecure when I see it online and I do not have it. Because of it I am working out to lose weight and to be as close as what I see in the media. I do not see it a bad thing for me because I only become aware of what I need to change and I know I can get it when I work for it. (Body Goals, 20)

So in our sports, and also in what they say, that if you’re big, you should be strong. (Throwing Events, 23)

It makes me feel like I need to work out. I shouldn’t eat today, right? I should balance my food. It influences the way I see myself. (Pilates, 23)

Swimming mentions that during a period of illness and weight loss, their teammates made comments about their physique. Despite this, Swimming chose not to criticize their teammates’ physiques in return. This shows that societal perceptions and comments can influence how we view our own bodies, and how we choose to respond to those comments. The influence of societal norms and expectations on our perceptions of our own bodies is a key aspect of body image. Body Goals is influenced by media depictions of body image, leading to feelings of insecurity when they perceive a discrepancy between their own body and those they see online. This can motivate them to engage in weight management behaviours, such as working out, in an attempt to reduce this perceived discrepancy. However, they also demonstrate a level of media literacy, recognizing that these images are often unrealistic and that striving to match them exactly may not be healthy or achievable.

Throwing Events discusses the societal expectation in sports that being bigger equates to being stronger. This is a common stereotype that can impact how athletes view their bodies and their performance. It shows how societal beliefs and expectations can shape our body image. Pilates explained that they feel a need to work out and manage their food intake, likely influenced by their body image and how they perceive themselves in relation to societal standards of health and attractiveness. This internalized pressure can motivate weight management behaviours, as individuals strive to achieve a body that they perceive as acceptable or desirable. In all these interview answers, body image serves as a significant motivator in managing weight. These participants highlight the impact of body image on weight management, emphasizing the importance of fostering a positive body image for a healthier approach to weight management.

Social norms:

Especially, when people normalize it. What the media normalizes is when you’re macho or sexy, you’re easily an 8 or a 10. (Swimming, 22)

Discipline comes from within, and social norms act as a boost to encourage you to put in more effort to lose weight. (Body Building, 19)

Of course, it is not new to us that there are social norms that consider only petite women as beautiful. It affected me severely especially when I was young, though I still often find myself being insecure especially with my legs because they say it is more thick than usual. And of course, in our country, it is not viewed as beautiful so I always question myself if I still fit into societal norms. (Shuawawa, 21)

For me, as a man, of course, as a man, as you can see before, Men do a lot. For example, in the old days, usually, for example, if you get water, if you cut a tree or something, men do all those. (Track and Field, 23)

Interviewee are influenced by societal expectations and norms, which shape their behaviours and attitudes. This is a key aspect of social norms. Swimming discusses how societal norms and media representations equate being “macho” or “sexy” with high attractiveness ratings. This shows how societal norms, particularly those perpetuated by media, can influence our perceptions and behaviours. In this case, the norm is that a certain body type or appearance is considered more attractive. Body Building talks about how discipline comes from within but also acknowledges that social norms can act as a boost to encourage weight loss efforts. This shows that while personal motivation is important, societal expectations (i.e., social norms) can also play a significant role in influencing behaviour. Shuawawa discusses the societal norm in her country that considers only petite women as beautiful. This norm has affected her self-perception, especially regarding her legs, and motivates her to question and potentially alter her physique to fit into these societal norms. Track and Field refers to traditional gender norms, where men are expected to be physically strong and active. This expectation, rooted in societal norms, motivates him to maintain his physical fitness and manage his weight.

Media influence:

With how the media depicts body image I think that its negative for many people as well as for myself. I get insecure when I see it online and I do not have it. Because of it I am working out to lose weight and to be as close as what I see in the media. I do not see it a bad thing for me because I only become aware of what I need to change and I know I can get it when I work for it. (Body Goals, 20)

For me, sometimes the media could be cruel in a sense, like in depicting body image. But personally, it does not affect how I manage my weight, like the strategies I use because I’m still running my own path and then other people don’t understand it. So as long as I run my own path in the weight management, I could care less about how the media depicts body image. (Basketball, 23)

So, yes. Until my weight loss became unhealthy. So, that’s one of the negative impacts of the media. And we should… We need to be aware of those things. Because, for example, I don’t realize that I’m adapting what they’re doing just to lose weight. (Fit Tracker, 22)

I’m glad that nowadays, because of the advent of technology, there’s a bunch of YouTubers and fitness influencers that I can rely on to improve my workouts. They show the reality that after the gym pump (the tight feeling in your muscles, and it feels like it is bigger) and without the gym lights, you’d look like a normal person and that progress takes time. (Angela, 22)

Body Goals is influenced by media depictions of body image, which leads to feelings of insecurity. This motivates them to work out and lose weight to align their physique with what they see in the media. The Basketball participant acknowledges the potential negative impact of media on body image but maintains their own path in weight management, showing resilience against media influence. The Fit Tracker user highlights the potential harm of media influence, as it led to unhealthy weight loss. This underscores the need for awareness and critical consumption of media content. Lastly, Angela appreciates the positive influence of fitness influencers on social media platforms, who provide realistic portrayals of fitness progress and motivate her to improve her workouts. These illustrate how media influence can serve as both a positive and negative motivator in managing weight, emphasizing the importance of media literacy in the context of weight management

## Health conciousness

For the third research question “What role does health consciousness play in an individual’s decision to manage their weight?”The theme health conciousness emerged in the analysis which then consisted of two codes (Priority of Health, and Health Concerns). Which indicates that these two are also deep motives for weight management.

Priority of health:

I feel that number one is health. Then, next is appearance. In other words, health is higher than appearance. (Track and Field, 23)

I prioritize my health more than my appearance. (Body Building, 19)

So, I started going to the gym so that I don’t get tired easily. So, I don’t get tired easily. And, I feel like I’m regaining my energy. And, I don’t feel heavy every day. So, that’s when I realized that it is also important to take care of your health, especially your weight. (Fit Tracker, 22)

The only issue I’m aware of is obesity. Yeah, obesity. Diabetes or certain foods, certain consumption habits that leads to … I’m not super-duper aware of them, but it just makes me conscious every once in a while to put restrictions within myself. So, if I eat too much sugar, stop it. Don’t eat sugar the next day. If I eat too much of this, don’t eat it the next day and stop. (Pilates, 23)

Each individual are expressing a preference for health over appearance, indicating that they value their well-being and fitness more than their physical looks. This is the essence of the “priority of health” concept. Track and Field participant places health above appearance, indicating that their primary motivation for weight management is to maintain or improve their health. Similarly, Body Building participant prioritizes health over appearance, reinforcing the idea that health is a key motivator. Fit Tracker started going to the gym to avoid fatigue and regain energy, showing that improving physical health and stamina is their motivation for weight management. Lastly, Pilates participant is aware of health issues like obesity and diabetes and adjusts their consumption habits accordingly to maintain their health. These responses highlight that the priority of health rather than societal or aesthetic standards, can be a strong intrinsic motivator in managing weight.

Health concerns:

So, I think my health concerns when I was in grade 9. In grade 9, I already had a mild pneumonia. So, it was very down for me as an athlete. I was a swimmer and I also had pneumonia. (Swimming, 22)

I do have asthma so I started working out to strengthen my body. Before because I am much more active with working out I do not get asthma attacks but now that I am starting to become too comfortable again. I am losing strength and stamina which now is making my asthma comeback. (Body Goals, 19)

So, I used to have anemia because of how often I’d starve myself or eat strictly 800 calories, but I’d still eat junk food—I just counted the individual chips that I ate via a calorie tracker. And I’d faint a lot because of it, and I was so happy because that meant I wouldn’t gain weight. But that also meant I was very weak. (Angela, 22)

And now, I have a PCOS. So, when you have PCOS, you gain weight. And it is more about water retention. So, it is one of the reasons why I decided to go to the gym to sweat so that the water won’t retain in my body. (Fit Tracker, 22)

Swimming mentions having had mild pneumonia in grade 9, which was a significant setback for them as an athlete and swimmer. Pneumonia is a serious health concern that can severely impact physical capabilities, especially for athletes where optimal lung function is crucial. Body Goals discusses their asthma, a chronic condition that influences their lifestyle. They mention that working out helped them manage their asthma and prevent attacks. However, they also note that becoming less active has led to a decrease in strength and stamina, and a return of their asthma symptoms. Angela experienced anaemia due to extreme dieting practices, which led to physical weakness. This negative health impact likely serves as a motivation to manage her weight in a healthier manner. Lastly, Fit Tracker has Polycystic Ovary Syndrome (PCOS), a condition often associated with weight gain and water retention.These health concern motivates them to exercise regularly to manage their weight. It illustrate how health concerns can serve as a significant motivator in managing weight.

## Past experiences in weight management and family support

With the last research question about “How does an individual’s family support and past experiences shape their motivation for weight management?” The theme Family Support and Past weight management experiences which then consisted of two codes (Learned Habits and Supportive Environment). These two also indicates that they are deep motives for weight management.

Learned habits:

Before I tend to not gain a lot of weight even if I eat a lot and also exercises a lot so that probably makes me even more hungry. But lately I seem to gain weight without even doing or eating that much. So now I’ve learned to be more balanced about it. I try to work out equivalent with what I ate. Just to burn enough and not to over workout. (Body Goals, 20)

At first, I only knew about going to the gym because you burn calories. But now, diet is more of a priority than working out. You might burn a lot, but if you keep consuming fats and calories, it is pointless. (Body Building, 19)

I tried. I made my own diet back then. I made it myself. it is not really… Effective? Or what not. I think it was effective, but then it wasn’t really super sustainable. (Pilates, 23)

I don’t want to go back if I look at my past self now, I don’t want to go back to that because it is draining. And it is hard to maintain that you don’t eat anymore just to maintain that figure, that appearance to other people. So now, I’m healthier. Although I try to lose weight, my approach is healthier. I seek help from my trainer on what I should eat. At least I still eat. But it is just a deficit. it is not unhealthy anymore like before. (Fit Tracker, 22)

Body Goals describes a change in their body’s response to food and exercise over time. Initially, they could eat and exercise a lot without gaining weight, but recently they’ve noticed weight gain even without significant changes in diet or activity. As a result, Body Goals has adjusted their habits to achieve a more balanced approach, matching their workout intensity with their food intake. This is a learned habit because it involves adapting behaviour based on personal experience and observed outcomes. Body Building initially believed that going to the gym and burning calories was the key to weight management. However, over time, they realized that diet plays a more crucial role. Despite the effort put into workouts, consuming high amounts of fats and calories can negate the benefits. This understanding led Body Building to prioritize diet over working out, which is a significant shift in behaviour and a clear example of a learned habit. Pilates experimented with their own diet, learning from the experience that it wasn’t sustainable. They then adapted a better approach to dieting based on this learning. Fit Tracker had a past habit of extreme dieting to maintain a certain appearance. They learned from this draining experience and now follow a healthier approach, seeking advice from a trainer on what to eat.

These interview answers highlighted that they have adjusted their behaviours based on their experiences and observations. They’ve learned from their past actions and used that knowledge to form new habits that better serve their goals. This process of learning and adaptation is at the heart of what is called “learned habits”.

Supportive environment:

In my family, I’m more motivated because I take criticism from people that I know that would make me better. (Swimming, 22)

Yeah, I can say they’re very supportive. For me, whatever I need, they’ll give it to me. So for example, if I only need chicken breast for this time, they’ll give it to me. So they’re really a big help. (Basketball, 23)

I feel like, in the family, they don’t have much. They just support my decision. Then, my decision, of course, I consult it to other coaches, teammates, people, friends. Of course, after that, I choose the best. After that, I tell my family. Then, when you tell them, they understand and support it. (Track and Field, 23)

My father is an instructor, so I have this awareness also about weight that it is not always too late to lose weight when you’ve gain weight. You just have to work out again and put the effort for it. Because of that I also believe that even if I gained weight now, it is not too late to get to my prime. (Body Goals, 20)

Swimming mentions that they are more motivated because they take criticism from people in their family who they believe can help them improve. This shows that their family provides an environment where constructive criticism is used as a tool for personal growth and betterment. This kind of environment can be very supportive as it encourages self-improvement and fosters resilience. Basketball describes their family as very supportive, providing whatever they need. In the context of Basketball’s fitness journey, their family’s support extends to dietary needs, such as providing specific foods like chicken breast. This kind of tangible support not only makes it easier for Basketball to stick to their fitness regimen but also shows a high level of understanding and encouragement from their family. Track and Field consults with coaches, teammates, and friends before making decisions about their weight management. They then communicate these decisions to their family, who provide understanding and support. This highlights the importance of having a supportive network that respects and supports one’s decisions. Body Goals has a father who is an instructor and provides them with awareness and advice about weight management. This familial support motivates them to put in the effort to lose weight and maintain their prime condition.

These individuals benefit from a supportive environment that facilitates their personal goals—in this case, related to fitness and health. Support can come in various forms, from emotional support and motivation to tangible assistance with specific needs.

## Discussions

The analysis of the interviews has revealed three candidate themes that, while not robust enough to be considered primary themes, exhibit significant relevance and warrant further exploration. These themes—Negative Self-Comparison, Unsupportive Environment, and Self-Acceptance—each play a crucial role in the participants’ experiences and motivations related to weight management.

Negative self-comparison emerged as a theme where participants compared themselves unfavourably to others, often leading to feelings of inadequacy and pressure to change. For instance, Pilates describes feeling pressured to manage her weight due to comparisons with her sister, a model. This aligns with existing literature suggesting that negative self-comparison can lead to both positive and negative outcomes in weight management behaviours. While it can motivate individuals to adopt healthier habits, it can also result in harmful practices and negatively impact mental health, leading to issues such as low self-esteem and body dissatisfaction (Festinger, 1954; Thompson et al., 1999). Therefore, while negative self-comparison can act as a motivator, it is essential for individuals like Pilates to approach weight management in a balanced and healthy manner.

The theme of an unsupportive environment was also prominent, with participants describing negative comments and lack of support from their surroundings as significant stressors. Angela recounts how discouraging remarks about her body changes made her feel pressured and demotivated. Similarly, Pilates shares how hurtful comments have diminished her self-esteem. This aligns with studies that highlight the impact of social environment on weight management, suggesting that negative feedback and lack of support can create psychological stress, potentially leading to unhealthy weight control practices (Puhl & Heuer, 2009). The pressure to conform to societal expectations, exacerbated by an unsupportive environment, underscores the need for positive reinforcement and supportive communities in facilitating healthy weight management.

Conversely, the theme of self-acceptance emerged as a positive and potentially empowering factor in weight management. Participants like Basketball and Angela have highlighted the importance of self-love and internal validation over societal standards. This is consistent with findings that self-acceptance can enhance psychological well-being and promote sustainable, healthy weight management practices (Avalos et al., 2005; Tylka & Homan, 2015). Self-acceptance allows individuals to set realistic goals based on personal well-being rather than external pressures, leading to more positive outcomes in their weight management journey. For instance, Pilates expresses a desire to embrace self-love and find happiness in her current state rather than postponing it.

The candidate themes identified in this study provide valuable insights into the complexities of weight management motivation. Negative self-comparison, while sometimes motivating, can also have detrimental effects on mental health. An unsupportive environment can exacerbate stress and hinder healthy practices, highlighting the need for positive social support. On the other hand, self-acceptance appears to foster a healthier and more sustainable approach to weight management. Future research should delve deeper into these themes to better understand their implications and to develop interventions that promote healthy weight management behaviours in supportive and self-affirming contexts.

In the analysis of the main themes, the thematic analysis has revealed that psychological motivation, particularly intrinsic and extrinsic factors, significantly influences individuals’ commitment to weight management. Extrinsic motivation emerges clearly in the responses of Track and Field, Throwing Events, Fit Tracker, and Pilates. These individuals are motivated by external factors such as team acceptance, performance enhancement, societal acceptance, and admiration from others. This aligns with Self-Determination Theory (SDT), which acknowledges that extrinsic motivators can play a role in driving behaviours, especially when they are internalized to some extent (Deci & Ryan, 2000). For example, the Track and Field athlete’s desire to fit in with their team mirrors the external pressures that can shape motivation, as highlighted by Teixeira et al. (2011), who note that external pressures can sometimes be integrated into one’s personal values, leading to sustained behaviour changes. However, these external motivators can be less effective for long-term adherence compared to intrinsic motivation, as they may not fulfill the fundamental psychological needs of autonomy, competence, and relatedness necessary for sustained motivation (Santos et al., 2016).

In contrast, intrinsic motivation is evident in the responses from the Body Building, Swimming, Shuawawa, and Angela participants. These individuals emphasize personal discipline, mental strength, personal satisfaction, and a desire for self-liberation as their primary drivers for weight management. According to SDT, intrinsic motivation arises when individuals engage in behaviours out of genuine interest and personal value, leading to more sustainable and self-determined actions (Deci & Ryan, 2000). The Body Building participant’s focus on discipline over fleeting motivation underscores the importance of internal commitment, resonating with Silva et al. (2010), who found that autonomy-supportive environments that respect individual choices foster intrinsic motivation. Similarly, the Swimming participant’s proactive mindset and emphasis on mental sacrifice align with the findings of Linde et al. (2006), where higher self-efficacy was associated with successful weight management. The intrinsic satisfaction described by Shuawawa, and Angela’s drive to break free from external expectations, further highlight the deep personal rewards that come from intrinsic motivation, which is crucial for long-term adherence and psychological well-being (Teixeira et al., 2011).

Moreover, the concepts of self-efficacy and locus of control are reflected in these motivations. The Body Building and Swimming participants exhibit high self-efficacy, believing strongly in their ability to achieve their weight management goals through personal effort and sacrifice (Bandura, 1977). This aligns with the findings of Napolitano et al. (2013), who demonstrated that self-efficacy-enhancing strategies lead to better weight loss outcomes. Angela’s narrative, involving a desire to reject external expectations, reflects an internal locus of control, where she attributes her success to her own actions rather than external factors (Rotter, 1966). This internal locus of control has been associated with greater motivation and proactive health behaviours, as noted by Wallston et al. (1978). Social support, another critical factor in weight management, is subtly present in the extrinsic motivations discussed. The Track and Field athlete’s need to fit in with their team and the Fit Tracker user’s desire for societal acceptance suggest that social contexts and external validation significantly influence their motivations. Wing and Jeffery (1999) found that social support from friends and family can enhance adherence to weight management programmes, highlighting the role of external factors in motivating individuals.

In summary, the interview contents demonstrate the complex interplay between extrinsic and intrinsic motivations in weight management. While extrinsic motivations, influenced by social pressures and external rewards, play a significant role, intrinsic motivations driven by personal values, self-efficacy, and internal locus of control are critical for long-term success. These findings align with the theoretical frameworks of SDT, self-efficacy, and locus of control, emphasizing the need for weight management interventions to foster intrinsic motivation and supportive environments that enhance self-efficacy and internal locus of control for sustainable outcomes.

The theme of societal influence, encompassing body image, social norms, and media influence, reflects the complex interplay between individuals and societal expectations.

The influence of body image on weight management is evident in how individuals respond to societal and peer feedback about their physiques. For instance, the Swimming participant noted how comments from teammates about weight loss during illness affected their self-perception, despite choosing not to criticize others in return. This highlights how societal perceptions can shape our body image and influence our reactions (Perloff, 2014). Similarly, Body Goals expressed feelings of insecurity due to media depictions of ideal body types, motivating them to engage in weight loss activities to reduce this discrepancy. This aligns with findings from Neumark-Sztainer et al. (2006), who highlighted that body dissatisfaction often drives weight management behaviours. Additionally, Throwing Events emphasized societal expectations in sports, where larger body sizes are equated with strength, illustrating how cultural stereotypes influence body image and weight management practices (Swami, 2015). The Pilates participant’s experience further supports this, as societal standards lead them to regulate their diet and exercise routines to align with perceived ideals (Grabe et al., 2008). These examples underscore the significant role of body image as a motivator for weight management, driven by societal perceptions and expectations.

Social norms also play a crucial role in shaping weight management behaviours. The normalization of specific body types, as mentioned by the Swimming participant, reflects how societal standards of attractiveness are deeply ingrained and perpetuated through media (Tiggermann & Slater, 2013). Body Building noted that while personal discipline is essential, social norms act as an additional motivator to pursue weight loss, demonstrating the interplay between individual motivation and societal expectations. This is consistent with Festinger’s (1954) Social Comparison Theory, which posits that individuals evaluate themselves against societal standards, influencing their motivation to conform (Myers & Crowther, 2009). Shuawawa’s account of societal norms favouring petite women reveals the pressure to conform to cultural ideals of beauty, impacting self-esteem and motivating weight management efforts. Track and Field highlighted traditional gender norms, where men are expected to be physically robust, further illustrating how social expectations drive weight management behaviours. These examples demonstrate that social norms significantly influence individuals’ motivations to manage their weight, often reinforcing specific body ideals.

The impact of media on body image and weight management is profound, as illustrated by several participants. Body Goals and Fit Tracker highlighted how exposure to idealized body images in media led to feelings of insecurity and unhealthy weight loss behaviours. This is corroborated by research indicating that media depictions of thinness as the ideal body type exert substantial pressure on individuals to engage in weight management practices (Fardouly et al., 2015; Rodgers et al., 2013). On the other hand, Basketball maintained resilience against media influence, focusing on personal goals rather than societal expectations. This perspective underscores the importance of media literacy and the ability to critically evaluate media content (Grabe et al., 2008). Angela’s positive experience with fitness influencers who provide realistic portrayals of fitness progress highlights how media can also serve as a motivational tool when it promotes healthy and achievable standards (Neumark-Sztainer et al., 2006). These varied responses to media influence illustrate its dual role as both a positive and negative motivator in weight management, emphasizing the need for critical engagement with media.

The societal standards and perceptions regarding body image play a pivotal role in influencing individuals’ motivations for weight management. The significant impact of media portrayal, societal expectations, and cultural ideals contributes to the formation of body dissatisfaction, which is closely linked to the motivation to partake in weight management behaviours (Neumark-Sztainer et al., 2006; Perloff, 2014). The Social Comparison Theory further elucidates how individuals assess their bodies in relation to others, driving their efforts to conform to societal norms (Myers & Crowther, 2009). Addressing these influences by fostering unfiltered body positivity and emphasizing health over appearance can help individuals develop a healthier relationship with their bodies and weight management behaviours (Neumark-Sztainer et al., 2006). The diverse experiences of the participants highlight the complex interplay between societal perceptions and individual motivations, underscoring the need for a nuanced approach to promoting unfiltered positive body image and healthy weight management practices.

The role of health consciousness in weight management is underscored by individuals’ awareness and prioritization of their health and well-being. Health consciousness, characterized by a deep concern for personal health and preventive behaviours (Hong, 2009), is crucial in motivating individuals to engage in weight management practices. According to the Health Belief Model (Rosenstock, 1974), perceived susceptibility to health risks drives individuals to adopt behaviours that prevent negative health outcomes. This model helps explain why health consciousness plays a significant role in individuals’ decisions to manage their weight.

Participants consistently expressed that maintaining health was more important than appearance. For instance, Track and Field emphasized placing health above appearance, indicating that the primary motivation for weight management is to sustain or enhance health. Similarly, Body Building echoed this sentiment, prioritizing health over looks. Fit Tracker went to the gym to avoid fatigue and regain energy, highlighting the importance of physical health and stamina. Pilates was aware of the health risks like obesity and diabetes and adjusted their consumption habits accordingly. This demonstrates a proactive approach to health, where the priority of maintaining well-being drives their weight management efforts. This aligns with findings by Puhl and Heuer (2009), who noted that health-conscious individuals are more likely to maintain their weight management efforts over time, underscoring the intrinsic motivation derived from valuing health over appearance.

Specific health issues also significantly influenced individuals’ motivations for weight management. Swimming participant recounted a setback due to mild pneumonia, emphasizing how serious health conditions can impact physical capabilities and motivate health-conscious behaviours. Similarly, a participant with the pseudonym Body Goals mentioned asthma and how regular workouts helped manage their symptoms, highlighting the direct relationship between physical activity and health management. Angela’s extreme dieting led to anaemia, causing physical weakness, which likely motivated her to adopt healthier weight management practices. Lastly, a Fit Tracker with Polycystic Ovary Syndrome (PCOS) described how regular exercise helps manage weight and symptoms associated with the condition. These personal health challenges serve as powerful motivators for engaging in weight management behaviours, as individuals seek to mitigate their health concerns through proactive measures. This aligns with research by Jayanti & Burns (1998), which suggests that individuals with high health consciousness are more likely to engage in preventive health behaviours, including weight management.

Moreover, the research by Cai et al. (2022) highlights that health consciousness significantly predicts dietary self-regulation, leading to better weight control outcomes. This supports the idea that fostering health consciousness can enhance dietary behaviours, contributing to effective weight management. The systematic review and meta-analysis by Madigan et al. (2022) further reinforce this by demonstrating that behavioural weight management interventions in primary care settings led to significant weight loss and waist circumference reduction, particularly among health-conscious individuals.

In conclusion, health consciousness, through the prioritization of health and response to health concerns, emerges as a crucial factor in motivating weight management behaviours. By valuing health above appearance and responding proactively to health issues, individuals are more likely to engage in and sustain weight management practices. This comprehensive approach not only addresses weight concerns but also promotes overall well-being, making health consciousness a vital component of effective weight management strategies (Hong, 2009; Madigan et al., 2022; Puhl & Heuer, 2009; Cai et al., 2022; Jayanti & Burns, 1998).

The excerpts provided shed light on the significant influence of both learned habits and supportive environments in shaping individuals’ motivations for weight management. Learned habits, as exemplified by the experiences of Body Goals, Body Building, Pilates, and Fit Tracker, underscore the pivotal role of personal experiences in shaping behaviours towards weight management (Wadden et al., 1983). Body Goals’ adaptation to a more balanced approach to diet and exercise reflects a learned habit developed in response to changes in their body’s response to food and activity levels. Similarly, Body Building’s shift in prioritizing diet over exercise demonstrates a fundamental change in behaviour influenced by past experiences and observations (Ogden, 2000). Pilates’ recognition of the unsustainability of their previous dieting approach highlights the importance of learning from past failures to inform future strategies (Wing & Phelan, 2005). Fit Tracker’s transition from extreme dieting to a healthier approach signifies a positive behavioural adaptation influenced by past struggles and a desire for sustainable change.

Furthermore, the excerpts emphasize the crucial role of a supportive environment, as illustrated by the experiences of Swimming, Basketball, Track and Field, and Body Goals. Swimming’s motivation derived from constructive criticism within the family underscores the importance of supportive feedback in fostering personal growth and resilience (Sallis et al., 2009). Basketball’s family’s tangible support in providing specific dietary needs demonstrates how familial support can facilitate adherence to healthy habits (M. L. Wang et al., 2014). Track and Field’s consultation process with various stakeholders followed by familial understanding and support highlights the importance of a supportive network that respects individual decisions (Trost et al., 2002). Moreover, Body Goals’ familial influence through their father’s advice and encouragement reinforces the positive impact of familial support in fostering motivation and determination for weight management (Gorin et al., 2014).

In essence, these findings align with existing research emphasizing the interplay between learned habits and supportive environments in shaping motivations for weight management. Individuals’ past experiences inform their behaviours and attitudes towards weight management, with successful adaptations often stemming from learned lessons and failures (Epstein et al., 2007). Additionally, the presence of a supportive environment, particularly within the family unit, can significantly enhance individuals’ motivation and adherence to healthy behaviours (Black et al., 2010). By recognizing the importance of both learned habits and supportive environments, interventions aimed at promoting healthy weight management can be tailored to address individual needs and foster a holistic approach towards sustainable behaviour change (Ball et al., 2010).

To end, based on this study, future research can apply and extend Self-Determination Theory (SDT) to weight management. A more comprehensive understanding could be gained by studying how autonomy, competence, and relatedness directly affect sustained behaviour change and weight control effectiveness. Explore potential moderating factors that may affect SDT-based therapies’ effectiveness, such as personality variations or cultural effects, to refine the theoretical framework. Practical applications require more research to develop and evaluate specific solutions based on this study’s different motives. Intervention strategies that explicitly address intrinsic and extrinsic motivations, cultural pressures, health consciousness, and family dynamics may be more effective. Practitioners and policymakers would benefit from longitudinal studies on the long-term effects of such treatments on weight management and well-being. In line with the changing healthcare and wellness landscape, studying the efficacy of adding technology like mobile apps or virtual support networks to weight control programmes may be a promising research topic.

## Conclusion

The Self-Determination Theory (SDT) provides a valuable framework for understanding how autonomy, competence, and relatedness drive sustainable behavioural changes in weight management.

Candidate themes presented possible topics to explore in future research although caution should be taken.

Intrinsic motivation plays a crucial role in long-term success, while extrinsic motivation can also contribute, particularly in the short term. The distinction between intrinsic and extrinsic motivations is highlighted, with intrinsic factors such as personal satisfaction and mental strength contrasting with extrinsic factors like societal acceptance and admiration from others. Self-Determination Theory (SDT) provides a lens through which to understand these motivations, emphasizing the importance of internalized extrinsic motivators and intrinsic motivators and the fulfilment of psychological needs for sustained behaviour change.

Societal influences, such as body image, social norms, and media influence, impact individuals’ perceptions and motivations regarding weight management. Societal influences, encompassing body image, social norms, and media influence, significantly impact individuals’ motivations for weight management. These highlights the need to address societal standards and promote unfiltered body positivity, sustainable standards and less extreme expectations that are not limited by internalized societal standards.

Family support and past weight management experiences shape individuals’ behaviours and provide valuable support in weight management journeys. Moreover, the role of learned habits emerges as a critical determinant of behaviour in weight management. Individuals adapt their behaviours based on past experiences and observations, emphasizing the need to learn from failures and successes to inform future strategies. Supportive environments, particularly within the family unit, provide essential social and tangible support that fosters motivation and adherence to healthy behaviours.

Health consciousness, driven by awareness and concern for well-being, influences decisions related to weight management efforts. Health consciousness emerges as a central motivator for weight management, driven by individuals’ prioritization of health and proactive response to health concerns. The Health Belief Model and related research underscore the importance of perceiving susceptibility to health risks in motivating preventive behaviours and sustaining weight management efforts.

Future research should extend SDT to explore how autonomy, competence, and relatedness directly affect sustained behaviour change and weight control effectiveness.

Consideration of moderating factors like personality variations and cultural effects can refine theoretical frameworks and intervention strategies.

Intervention strategies addressing intrinsic and extrinsic motivations, cultural pressures, health consciousness, and family dynamics may enhance effectiveness in weight management programmes.

Longitudinal studies are needed to understand the long-term effects of interventions on weight management and overall well-being.

Exploring the integration of technology, such as mobile apps or virtual support networks, into weight control programmes could be a promising avenue for future research and practical applications.

## Data Availability

The participants of this study did not give written consent for their data to be shared publicly, so due to the sensitive nature of the research and the need to uphold the anonymity of the participants, supporting data is not available.
